# The Human Hyoid Body–Greater Cornu Junction: Development, Histological Diversity, and Imaging Correlates

**DOI:** 10.7759/cureus.115209

**Published:** 2026-08-26

**Authors:** Konstantinos Bastalias, George Triantafyllou, Alexandros Samolis, Dimosthenis Chrysikos, Christos Lyrtzis, Nikolaos Lazaridis, Theodore Troupis, Maria Piagkou

**Affiliations:** 1 Anatomy, Faculty of Health Sciences, National and Kapodistrian University of Athens, Athens, GRC; 2 Anatomy and Surgical Anatomy, Faculty of Health Sciences, Aristotle University of Thessaloniki, Thessaloniki, GRC

**Keywords:** embryology, histology, hyoid bone, ossification, synchondrosis, synostosis

## Abstract

The human hyoid develops from anatomically distinct components, yet in anatomical, radiological, and forensic reports, the body-greater cornu junction is often reduced to a binary fused/unfused state. This structured narrative review with critical appraisal evaluates human evidence concerning embryological development, postnatal ossification, histological organization, age-related junctional union, and imaging interpretation of this interface. PubMed/MEDLINE and Google Scholar were searched through July 2026 using combinations of terms related to the hyoid bone, development, embryology, ossification, fusion, nonfusion, synostosis, synchondrosis, histology, and computed tomography; reference lists of relevant studies were also examined. Human serial-embryological, cadaveric, histological, radiographic, CT, three-dimensional CT, and micro-CT studies were prioritized. Because study populations, definitions of fusion, age strata, imaging resolution, and side-level versus person-level units of analysis were insufficiently harmonized, the evidence was synthesized narratively rather than pooled quantitatively. Direct human embryological observations support a spatially distinct median hyoid-body condensation but do not establish cellular lineage. Postnatal ossification of the body and greater cornua is distinct from subsequent remodeling and possible union of their interface. In adults, the junction may remain cartilaginous or joint-like, exhibit peripheral mineralization, develop partial osseous bridging, or undergo complete synostosis. These configurations represent distinct observed states and should not be assumed to constitute an obligatory longitudinal sequence. Junctional union becomes more frequent with age, but complete synostosis may occur in young adults, whereas nonfusion may persist into advanced age. Radiographic approximation, sclerosis, or marginal mineralization should therefore not be equated automatically with histologically complete osseous continuity. A provisional four-category operational framework is proposed to improve consistency across anatomical, imaging, and forensic studies. Future research should prioritize same-specimen correlation of standardized thin-section CT, micro-CT, and histology in independently documented, age-balanced human material.

## Introduction and background

The hyoid is a mobile, U-shaped bone situated between the mandible and laryngeal skeleton. Through its muscular, fascial, and ligamentous attachments to the tongue, floor of the mouth, pharynx, larynx, mandible, clavicle, and sternum, it contributes to swallowing, phonation, airway stabilization, tongue movement, and mandibular function despite not articulating directly with another bone [1].

The adult hyoid comprises a body and paired greater and lesser cornua. Developmental and postnatal evidence indicates that the body and greater cornua form and ossify as distinct components [2-4]. Their intervening junction, however, remains structurally variable into adulthood and may contain hyaline or fibrocartilage, a cleft or joint-like space, peripheral mineralization, incomplete osseous bridging, or complete synostosis [5-8]. These configurations are not biologically equivalent. In particular, ossification of the individual components, remodeling or mineralization of the intervening junction, and complete osseous synostosis represent distinct biological observations and should be reported separately. This distinction has direct clinical and forensic relevance. A persistent body-greater cornu junction may resemble a fracture on projection radiography or CT, whereas narrowing, sclerosis, or peripheral mineralization may create an appearance of fusion despite persistence of nonosseous tissue [6, 7, 9]. Moreover, studies of age-related hyoid change have used the term “fusion” to refer to non-equivalent endpoints, ranging from radiographic approximation or graded junctional change to partial osseous bridging, unilateral person-level union, and complete bony continuity [6-8, 10-16]. Consequently, reported prevalence estimates and proposed age thresholds cannot be compared reliably unless the definition of fusion, imaging or histological method, laterality, and unit of analysis are specified.

The present article therefore examines the body-greater cornu junction as a structured narrative review with critical appraisal. Its objectives are to distinguish what can be established from human embryological, postnatal, histological, and imaging evidence; to determine how variation in the operational definition of fusion contributes to apparently conflicting age-, sex-, and laterality-related findings; to identify the principal limitations of the existing evidence; and to propose a transparent terminology framework for future anatomical, radiological, and forensic studies. The proposed terminology is intended as a provisional harmonization framework requiring future validation rather than as established or consensus nomenclature.

## Review

Methodology

Review Design and Information Sources

This article is a structured narrative review with critical appraisal, rather than a systematic or scoping review. The literature review was designed to identify and critically compare evidence directly relevant to the human body-greater cornu junction, with emphasis on developmental anatomy, postnatal ossification, histological organization, junctional fusion or synostosis, and imaging interpretation. It was not designed to generate a pooled prevalence estimate. PubMed/MEDLINE and Google Scholar were searched through July 2026, and the reference lists of relevant primary studies and review articles were examined to identify additional publications.

The PubMed search combined terms relating to the hyoid bone and body-greater cornu junction with terms addressing development, embryology, ossification, fusion, nonfusion, synostosis, synchondrosis, calcification, histology, and computed tomography. The principal concept set was: ("hyoid bone"[Title/Abstract] OR "hyoid body"[Title/Abstract]) AND (development [Title/Abstract] OR embryology [Title/Abstract] OR ossification[Title/Abstract] OR fusion[Title/Abstract] OR nonfusion[Title/Abstract] OR "non-fusion"[Title/Abstract] OR synostosis[Title/Abstract] OR synchondrosis[Title/Abstract] OR calcification[Title/Abstract] OR histology[Title/Abstract] OR "computed tomography"[Title/Abstract] OR CT[Title/Abstract] OR "micro-computed tomography"[Title/Abstract] OR imaging[Title/Abstract]).

Targeted Google Scholar searches combined “hyoid bone” or “hyoid body” with “greater cornu” or “greater horn” and corresponding terms relating to development, fusion, histology, and CT. Additional targeted searches were undertaken to identify recent studies addressing age, sex, fusion status, three-dimensional imaging, and postmortem imaging.

The search was not prospectively designed or documented in accordance with PRISMA guidelines. Record-level retrieval counts, duplicate-removal statistics, and exact day-level search dates were not preserved during the original literature review and have therefore not been reconstructed retrospectively. Accordingly, the article is not presented as a PRISMA-compliant systematic review, and no PRISMA flow diagram is provided.

Eligibility and Evidence Selection

Primary human studies were prioritized when they directly evaluated one or more of the following: (1) embryological formation of the hyoid body or greater cornu; (2) postnatal ossification of individual hyoid components; (3) histological composition of the body-greater cornu interface; (4) radiographic, CT, or micro-CT appearances of junctional nonfusion, mineralization, or fusion; and (5) associations of age, sex, or laterality with junctional union. Studies of general hyoid morphometry, three-dimensional shape, postmortem trauma, or forensic age or sex estimation were retained only when their findings contributed directly to interpretation of the body-greater cornu junction [14-21].

Non-human comparative studies and reports concerned exclusively with unrelated hyoid abnormalities or other regions of the hyoid apparatus were excluded from the core evidence synthesis. Findings from other hyoid junctions were not used to infer the developmental trajectory of the body-greater cornu interface. No language restriction was prespecified; older non-English anatomical studies were retained when sufficient methodological and results information was available to permit reliable interpretation [5].

Study selection was not performed through a prospectively registered, duplicate-independent systematic-review workflow. Publications were assessed for direct relevance to the predefined anatomical question and critically evaluated against the reported endpoint and method. This limitation is explicitly acknowledged and is one of the reasons the present article is classified as a structured narrative review rather than a systematic review.

Data Extraction, Critical Appraisal, and Synthesis

For each core study, the synthesis records, where available, the sample size and age range, study material, imaging or histological method, operational definition of fusion or synostosis, unit of analysis (junction/side or person), principal quantitative or qualitative findings, potential cohort overlap, and major methodological limitations.

Because the evidence base includes fundamentally different study designs-serial human embryology, cadaveric anatomy and histology, conventional radiography, clinical CT, three-dimensional CT, and micro-CT-a single numerical risk-of-bias instrument was not applied across all studies. Instead, critical appraisal focused on six domains: (1) sampling and age distribution; (2) definition of the anatomical endpoint; (3) imaging resolution or histological validation; (4) unit of analysis and treatment of paired sides; (5) control of age, sex, and other potential confounders; and (6) independence of the study sample from other published datasets.

No meta-analysis, meta-regression, pooled prevalence estimate, I² statistic, or τ² estimate was calculated. Quantitative pooling was considered inappropriate because the available studies differ substantially in their definitions of fusion, age stratification, study populations, imaging or histological methods, and use of side-level versus person-level denominators. In addition, the independence of some cadaveric histological datasets is uncertain [5-8]. The age-fusion relationship was therefore synthesized descriptively using study-specific frequencies, age ranges, thresholds, and statistical model findings, where reported.

Embryological construction of the hyoid

Traditional and Revised Developmental Models

The conventional anatomical teaching model attributes the lesser cornu and a cranial contribution to the hyoid body to the second pharyngeal arch, and the greater cornu and a caudal contribution to the body to the third arch [1, 2]. This model remains useful as a developmental schema, but it should not be regarded as equivalent to direct evidence of human cellular lineage.

**Figure 1:**
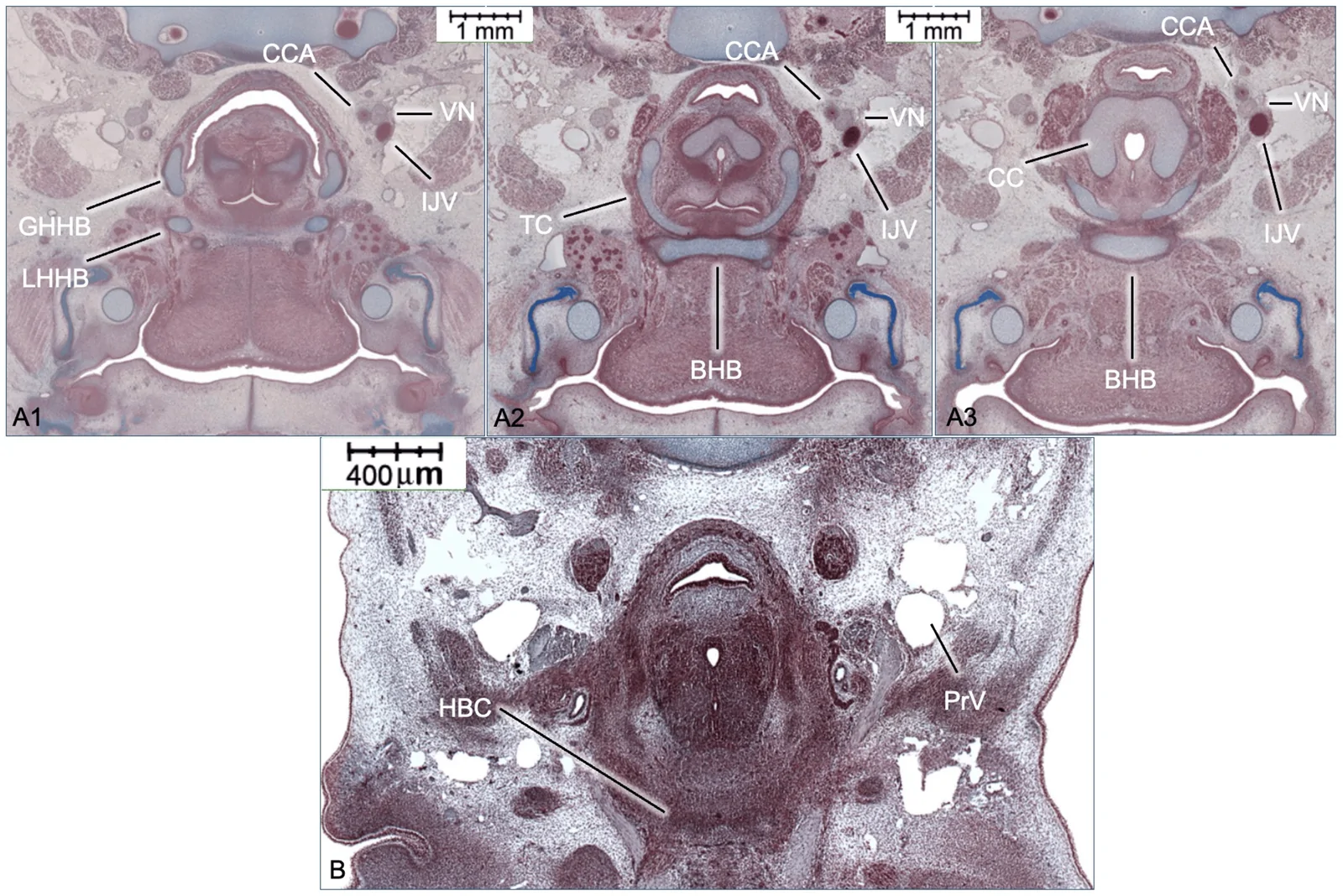
Developmental anatomy of the human hyoid bone. Axial histological sections of human embryos at Carnegie stage 23, approximately 56 days of development (A1–A3), and Carnegie stage 19, approximately 46 days of development (B). Panels A1–A3 represent sections at different craniocaudal levels. Panel A1 shows the lesser and greater cornua of the developing hyoid. Panel A2 shows the hyoid body in relation to the thyroid cartilage, and panel A3 shows the hyoid body in relation to the cricoid cartilage. Panel B demonstrates the median hyoid body condensation. BHB, body of the hyoid bone; CC, cricoid cartilage; CCA, common carotid artery; GHHB, greater cornu of the hyoid bone; HBC, hyoid body condensation; IJV, internal jugular vein; LHHB, lesser cornu of the hyoid bone; PrV, precardinal vein; TC, thyroid cartilage; VN, vagus nerve. Scale bars: 1 mm in A1-A3 and 400 μm in B. Images obtained and adapted from the open-access repository: Digitally Reproduced Embryonic Morphology (DREM) project (http://virtualhumanembryo.lsuhsc.edu).

Rodríguez-Vázquez et al. examined serial human embryonic and fetal material and described a solitary median mesenchymal condensation in the tongue-base region that was spatially distinct from the lateral second- and third-arch cartilaginous elements; cartilage subsequently developed within this median condensation [2]. The lesser cornu remained associated with Reichert cartilage, whereas the greater cornu was related to third-arch cartilage. Three-dimensional reconstructions by de Bakker et al. demonstrated a comparable spatial organization [3]. Together, these observations provide morphological evidence for a spatially distinct median body anlage and lateral greater-cornu precursor, but they do not establish cellular lineage or determine the subsequent biological fate of the postnatal body-greater cornu interface (Figure 1).

The embryological evidence therefore supports a developmental interpretation in which the body and greater cornu arise as spatially distinct components. It does not establish whether an adult cartilaginous, joint-like, mineralized, partially bridged, or completely synostosed junction represents a compulsory stage of maturation, a persistent anatomical phenotype, secondary remodeling, or some combination of these processes. Findings involving the lesser-greater cornu or stylohyoid interfaces are anatomically indirect and are therefore not used to infer the developmental trajectory of the body-greater cornu junction.

Related Junctional Development

Between approximately eight and 10 weeks of development, the mesenchymal continuity between the cranial and caudal segments of Reichert cartilage generally disappears [4]. Persistence of this continuity has been proposed as one possible explanation for continuous cartilaginous or ossified configurations of the stylohyoid chain, rather than attributing every such variant to secondary postnatal mineralisation of the stylohyoid ligament [4]. Although this process does not involve the body-greater cornu junction directly, it illustrates that embryonic connections within the hyoid and stylohyoid apparatus may persist or regress to different degrees [4].

Rodríguez-Vázquez et al. described a cleft or joint cavity between the lesser and greater cornua during fetal development [5]. This interface is anatomically distinct from the body-greater cornu junction and should not be considered direct evidence for the development of the latter. Its relevance is mainly comparative, as the variable timing of cavity formation suggests that junctional maturation within the hyoid may not occur according to a uniform developmental sequence [5]. The authors also discussed a possible influence of surrounding muscular or fascial relationships, although a direct mechanical effect on cavity formation has not been established [5].

Comparative Mammalian Context

The human hyoid represents a reduced form of the more extensive multi-element hyoid apparatus found in many mammals. In numerous placental species, the basihyal and paired thyrohyals are connected to the basicranium through additional ossified elements [6-8]. Comparative primate studies demonstrate substantial variation in basihyal morphology and in the retention or reduction of the ossified suspensory chain [6-8].

Anthropoid primates generally possess a broad basihyal and a reduced bony connection with the skull, whereas many nonanthropoid primates retain a more complete ossified chain [6]. CT-based research in dogs has also identified breed-related differences in the orientation and compactness of the hyoid apparatus [22]. These comparative findings should not be interpreted as evidence for a direct anatomical equivalent of the human body-greater cornu junction. Rather, they show that variation in the number, arrangement, and degree of osseous connection between hyoid elements is common among mammals [6-8, 22]. They therefore provide a broader anatomical context for understanding the variable continuity of components within the human hyoid apparatus.

Postnatal ossification is distinct from adult fusion

Ossification of the individual hyoid components begins well before possible osseous union of the body and greater cornua. In Reed’s radiographic series, hyoid ossification was detectable during late fetal life; the body and greater cornua became radiographically ossified during infancy, whereas ossification of the lesser cornua occurred substantially later [4]. These findings describe mineralization of individual skeletal components and should not be interpreted as evidence of body-greater cornu synostosis.

CT studies reinforce this distinction. Fisher et al. assessed each body-greater cornu junction separately and classified it as distant nonfusion, nonfusion, partial fusion, or fusion, defining distant nonfusion as a body-cornu separation greater than 2.5 mm [8]. Distant nonfusion was restricted to individuals younger than 20 years in their cohort, whereas closely apposed but nonfused junctions persisted into older adulthood [8]. Thus, age-related reduction in the distance between the components is not biologically equivalent to complete osseous continuity.

Ito et al. evaluated 600 individuals using three-dimensional CT and described age-related changes in ossification at the body-greater horn junction as one component of hyoid maturation [14]. Naimo et al. similarly demonstrated the utility of CT for evaluating hyoid development, anatomical variation, and trauma and documented persistence of nonfusion in some older individuals [15]. These imaging studies provide important information regarding age-associated mineralized morphology but cannot, in isolation, establish microscopic tissue continuity across the junction.

Histological diversity and the meaning of “fusion”

Histological States

Histological evidence demonstrates that a radiographically nonossified body-greater cornu junction does not correspond to a single tissue phenotype. Koebke and Saternus described hyaline-cartilaginous and fibrocartilaginous interfaces, residual cartilage, clefts, and joint-like spaces in adult hyoids [5]. Shimizu et al. subsequently documented age-related narrowing and ossification of the junction while demonstrating persistence of chondroid tissue in some morphologically advanced configurations [6].

Kanetaka et al. examined 483 evaluable junctions from 259 cadavers and classified them into three histological grades: Grade I, predominantly fibrocartilaginous; Grade II, peripheral calcification or ossification with persistence of separation between the body and greater cornu; and Grade III, true osseous synostosis, in some specimens accompanied by continuity of the medullary spaces [7]. The prevalence of Grade III increased from 14.1% in the youngest age group to 35.9% in the oldest group; nevertheless, most junctions in the oldest group still did not demonstrate complete synostosis [7]. These cross-sectional findings demonstrate an age-associated difference in the distribution of histological states but do not establish that individual junctions necessarily progress sequentially from Grade I through Grade III.

The Shimizu and Kanetaka studies also require caution when considered as independent evidence of prevalence. The reports share investigators and arise from closely related cadaveric and histological research, while the published information does not permit specimen-level determination of the extent of potential cohort overlap [6, 7]. Their concordant observations therefore strengthen the evidence for histological heterogeneity, but their prevalence estimates should not be interpreted as fully independent replication or combined quantitatively.

Four-category operational framework

To improve comparability across studies, four operational categories are distinguished in this review: (1) open/nonfused junction, defined by absence of osseous continuity between the body and greater cornu; (2) junctional mineralization without bridging, defined by calcification or peripheral ossification that does not establish a continuous bony connection; (3) partial osseous bridging, defined by incomplete bony continuity across only part of the interface; and (4) complete osseous synostosis, defined by continuous osseous bridging across the junction. These categories describe observable junctional morphology and should not be interpreted as obligatory sequential stages. Complete synostosis is most strongly supported on imaging when cortical and trabecular continuity can be demonstrated, whereas histology remains the reference standard for determining microscopic tissue composition and confirming residual cartilage or other nonosseous tissue [5-7].

This framework also separates side-level morphology from person-level laterality. An individual may demonstrate complete synostosis on one side while the contralateral junction remains open, mineralized without bridging, or partially bridged. Accordingly, unilateral fusion is a person-level descriptor and should not be combined directly with side-level prevalence estimates of fused junctions. Fisher et al.’s category of distant nonfusion is retained as a useful study-specific CT descriptor, defined by a body-cornu separation greater than 2.5 mm, rather than interpreted as a universal anatomical or developmental stage [8].

**Figure 2:**
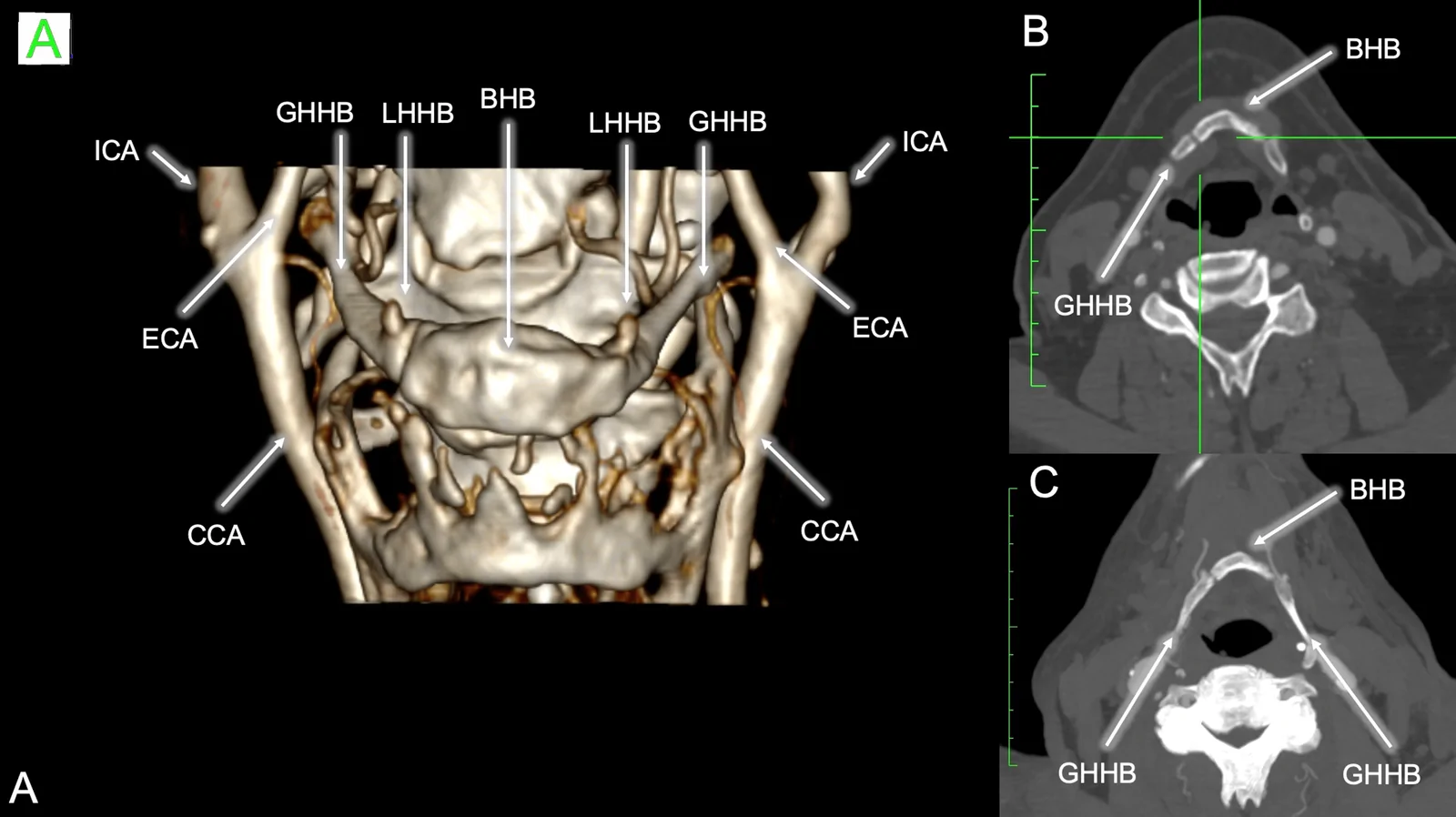
Computed tomography angiography of a 53-year-old man showing asymmetric body–greater cornu fusion, with persistence of the right body–greater cornu junction. (A) Anterior three-dimensional volume-rendered reconstruction showing the hyoid bone and its relationship to the carotid arteries. (B) Axial source image demonstrating the persistent right-sided body–greater cornu interface. (C) Axial maximum-intensity-projection reconstruction showing the hyoid body and both greater cornua. BHB, body of the hyoid bone; CCA, common carotid artery; ECA, external carotid artery; GHHB, greater cornu of the hyoid bone; ICA, internal carotid artery; LHHB, lesser cornu of the hyoid bone. Image Credit: Dr. George Triantafyllou.

Direct evidence, however, is lacking. No longitudinal human study has examined whether swallowing mechanics, muscle geometry, or local loading influence later synostosis, and no validated biomechanical model has established the effect of fusion on force transmission across the junction. The body-greater cornu interface should therefore be regarded as a developmentally distinct tissue junction whose morphology may be influenced by mechanical factors, rather than simply as an age-related marker (Figure 2, Table 1).

**Table 1:** Evidence domains at the body-greater cornu junction.

Domain	Typical observation	Principal evidence	What it establishes	What should not be inferred
Developmental process	Spatially distinct median body condensation and lateral greater-cornu precursor	Serial human embryology and 3D reconstruction [2,3]	Morphological separation during human development	Cell lineage, adult junctional fate, or obligatory fusion sequence
Histological state	Hyaline/fibrocartilage, cleft/joint-like space, marginal ossification, partial bridge, complete synostosis	Adult gross anatomy and histology [5-7]	Tissue-level diversity of the interface	That all states represent sequential stages in one person
Imaging appearance	Gap width, cortication, sclerosis/mineralization, partial or complete bony continuity	Radiography, CT, 3D CT, micro-CT [8,18-23]	Macroscopic mineralized morphology at the resolution of the modality	Precise cartilage type or microscopic synostosis when resolution is insufficient
Interpretation	Developmental junction versus trauma; side-level versus person-level fusion category	Cross-modal critical synthesis [5-8,23,24]	A reporting framework tied to modality and unit of analysis	A single universal chronological threshold for age estimation

Age, sex, laterality, and unit of analysis

Age-Related Variation: Substantial Overlap Rather Than a Fixed Cutoff

Most anatomical and imaging studies demonstrate an age-associated increase in body-greater cornu union, but the distributions of fused and nonfused states overlap substantially across adulthood. D’Souza et al. and Harjeet et al. reported no fusion within their youngest age strata while also documenting persistent nonfusion at considerably older ages [22-24]. Gupta et al. identified 87 bilaterally nonfused, 28 unilaterally fused, and 55 bilaterally fused hyoids among 170 specimens [25]. Balseven-Odabasi et al. reported bilateral fusion in 53 of 85 hyoids, although the age and sex composition of that sample limits direct comparison with larger CT-based cohorts [26]. Modern CT studies illustrate this overlap more clearly. Fisher et al. examined 136 CT studies across an age range of 1-94 years and classified individual junctions as distant nonfusion, nonfusion, partial fusion, or fusion [8]. No partial or complete fusion was observed before 20 years. Bilateral distant nonfusion, defined as a body-cornu separation greater than 2.5 mm, was present in 100% of individuals younger than 14 years and 50% of those aged 14-19 years, whereas bilateral nonfusion without a distant gap persisted in 16% of the oldest adult group [8]. Their statistical models further showed that fusion category and bone density contributed to age-group prediction in females, whereas neither variable significantly predicted age category in males [8]. These findings support an age association but argue against a deterministic chronological cutoff. Cundey et al. evaluated 220 multislice CT examinations in adults aged 18-92 years and documented bilateral fusion as early as 22 years and persistent nonfusion at 89 years [24]. A 2026 CTA study of 406 individuals aged 2-95 years similarly reported no fusion below 20 years, with the earliest bilateral fusion observed in a 22-year-old man and the earliest unilateral fusion in a 25-year-old woman [13]. Overall fusion proportions did not differ significantly between sexes, although unadjusted age distributions among individuals demonstrating fusion differed between males and females [13]. Mutlu et al., using three-dimensional CT in 320 individuals, likewise demonstrated an age-related increase in fusion while documenting persistence of nonfusion in older age groups [16]. Histological evidence provides an additional reason to avoid a binary chronological interpretation. In Kanetaka et al., the prevalence of Grade III true synostosis increased from 14.1% in the youngest age group to 35.9% in the oldest age group; nevertheless, most junctions in the oldest group still lacked complete synostosis [7]. Collectively, these findings support a probabilistic age-associated shift in the distribution of junctional states rather than an obligatory timetable culminating in complete synostosis.

Sex, Laterality, and Unit of Analysis

Reported associations between sex and body-greater cornu fusion are inconsistent. O’Halloran and Lundy reported more frequent fusion in males, whereas Miller et al. and several subsequent CT studies did not demonstrate a consistent overall sex association [8, 10, 11, 13, 15, 16]. Interpretation requires distinction between unadjusted sex differences in observed proportions and evidence for an independent sex effect after adjustment for age and other potential confounders.

Fisher et al. used multivariable models incorporating fusion category and bone density and identified sex-specific differences in their ability to predict age category [8]. These findings, however, do not demonstrate that sex itself is an independent determinant of complete synostosis. Most other studies were not designed to isolate an age-adjusted independent effect of sex. The available evidence therefore supports an absence of a consistently demonstrated association, rather than evidence that sex has no biological influence on junctional remodeling or synostosis. No consistent right-to-left sequence of fusion has been demonstrated. Fisher et al. found no significant side difference and analyzed the two junctions as paired side-level observations before deriving person-level categories [8]. Laterality findings vary across other studies [10, 11, 16, 22-26].

Future studies should therefore retain the right and left junctions as paired observations within each individual, report side-level morphology separately from person-level fusion patterns, and use statistical approaches that account for within-person dependence (Table 2).

**Table 2:** Core studies and methodological sources of heterogeneity.

Study	Sample/design	Modality	Operational fusion definition	Unit of analysis	Key quantitative contribution	Independence / principal limitation
Koebke et al. [5]	504 adult hyoids; histology in 49	Gross anatomy, radiography, histology	Historical anatomical/radiographic ossification plus tissue-level assessment; not equivalent to modern CT thresholds	Mixed specimen/junction reporting	Multiple tissue phenotypes; persistent nonossified interfaces in older adults	Independent historical series; terminology differs from modern imaging categories
Shimizu et al. [6]	238 cadavers; 31 histology	Contact radiography + histology	Degree of joint-space narrowing/ossification; histology showed residual chondroid tissue with peripheral ossification	Primarily junction/side	Age-related narrowing and increased fusion; imaging appearance not identical to tissue continuity	Possible overlap with Kanetaka series; extent cannot be verified
Kanetaka et al. [7]	259 cadavers; 483 evaluable junctions	Histology	Grade I fibrocartilage; Grade II peripheral calcification/ossification without union; Grade III true synostosis	Junction/side	Grade III 14.1% youngest to 35.9% oldest	Possible overlap with Shimizu; side-level denominator; cross-sectional
Gupta et al. [25]	170 excised hyoids, 20-65 y	Direct anatomical assessment	Person-level bilateral nonfusion, unilateral fusion, bilateral fusion	Person	87 bilateral nonfusion; 28 unilateral fusion; 55 bilateral fusion	Mortuary sample; limited oldest-age representation; endpoint less granular than histology
D’Souza et al. [22]	130 excised hyoids, 4-70 y	Radiography/anatomy	Presence of unilateral/bilateral union	Person + side descriptors	No fusion below 20; nonfusion after 60	Population-specific autopsy sample; broad binary/side categories
Harjeet et al. [23]	200 hyoids, 18-85 y	Radiography	Radiographic union of greater cornu with body	Person/side	No fusion before 25 in the study sample; persistent nonfusion in older adults	Population-specific; age strata and reporting differ from CT studies
Balseven-Odabasi et al. [26]	85 excised hyoids, 21-80 y	Dissection + morphometry	Bilateral/unilateral fusion on anatomical assessment	Person	53/85 reported bilaterally fused	Small age-sex subgroups; internal reporting inconsistency noted in original review
Fisher et al. [8]	136 CT, 1-94 y	Multiplanar CT + 3D	Side ranks: distant nonfusion (>2.5 mm), nonfusion, partial fusion, fusion; person categories derived bilaterally	Side first, then person	No partial/complete fusion <20; 100% distant nonfusion <14; 16% bilateral nonfusion in 75-95 y	Well-defined CT ranking and reliability; CT cannot establish microscopic tissue composition
Jadav et al. [12]	Autopsy-based radiological cohort	Radiography	Graded nonfusion, initiation/partial fusion, complete fusion	Side/person categories	Age-associated graded changes with overlap between stages	Uneven age/sex distribution; radiographic endpoint
Mutlu et al. [16]	320 clinical CT	3D CT	Degree of fusion categorized on CT	Person/side	Fusion increased with age; nonfusion persisted in older group; discriminant analysis explored age/sex	Population-specific; imaging thresholds not histologically validated
Cundey et al. [24]	220 MSCT, 18-92 y	Multiplanar CT + 3D	CT classification of fusion/nonfusion and morphology	Person + side descriptors	Bilateral fusion at 22; nonfusion at 89	Retrospective population-specific cohort; CT endpoint
Abbasoğlu et al. [13]	406 CTA, 2-95 y; 203 F/203 M	CTA	Nonfused, unilateral fusion, bilateral fusion on CT	Person	No fusion <20; first bilateral 22 M, unilateral 25 F; overall rates not significantly different by sex	Large balanced cohort; no histological validation; cross-sectional

Imaging-histology correlation

On projection radiography, the body-greater cornu junction may appear as a vertical or oblique radiolucent line and can resemble a fracture if its characteristic position and margins are not recognized [17]. CT improves anatomical localization, but its appearance can be affected by section thickness, reconstruction parameters, image plane, and display settings [17, 25, 27]. Three-dimensional surface rendering may assist spatial orientation, but it should not replace assessment of the axial and multiplanar source images, because a narrow residual junction may be less apparent on rendered images and apparent continuity may depend on the segmentation threshold.

Radiological interpretation should describe visible morphology rather than assign a histological diagnosis that exceeds the resolution of the modality. A smooth, corticated interface in the expected anatomical location, particularly without displacement or adjacent soft-tissue injury, is more suggestive of a persistent developmental junction than an acute fracture [17]. Narrowing, sclerosis, or partial mineralization may indicate junctional remodeling, but does not establish osseous synostosis [10, 11]. Uninterrupted cortical and trabecular continuity on multiplanar CT images is more consistent with complete osseous fusion. However, imaging alone may not always demonstrate the exact tissue composition of the junction, particularly when ossification is predominantly peripheral [10, 11].

High-resolution ex vivo methods offer greater detail for validation of imaging findings. Micro-CT can demonstrate thin mineralized bridges, cortical continuity, and trabecular architecture [12, 26], whereas contrast-enhanced CT may improve three-dimensional visualization of nonmineralized tissues [26]. Histology remains necessary to identify cartilage type, clefts, vascular invasion, mineralization, and marrow continuity [10, 11, 24]. Studies examining clinical CT, micro-CT, and histology in the same specimens would help determine which imaging appearances correspond to true osseous synostosis and which represent mainly peripheral mineralization (Table 3).

**Table 3:** Recommended terminology for body-greater cornu appearances.

Term	Operational meaning	Provenance/status	Recommended use	Critical caution
Distant nonfusion	Substantial body-cornu separation; Fisher et al. used >2.5 mm	Established study-specific CT term [8]	Developmental/age-related CT descriptor when threshold is stated	Do not assume the same threshold applies across scanners/reconstructions
Open/nonfused junction	No osseous continuity; components may be widely separated or closely apposed	Author-proposed harmonized term, informed by [5-8]	Side-level reporting with modality specified	Intervening tissue may be cartilage, fibrocartilage, fibrous tissue, or a joint-like space
Junctional mineralization without bridging	Calcification/peripheral ossification without continuous bony connection	Author-proposed harmonized term, informed by histology [6,7]	Imaging or histological descriptor	Not equivalent to partial or complete synostosis
Partial osseous bridging	Incomplete bony continuity across only part of the junction	Term used variably in imaging/histology; standardized here [7,8]	Use with stated modality and threshold	Peripheral mineralization should not automatically be called partial fusion
Complete osseous synostosis	Continuous osseous bridging across the junction; ideally cortical/trabecular continuity	Established anatomical concept; operationalized here [6,7]	Specify side, modality, and criteria	Routine CT may not prove microscopic continuity
Unilateral/bilateral fusion	Person-level pattern based on the two side-level junctions	Established person-level descriptor [8-20]	Report in parallel with side-level data	Do not mix person-level prevalence with side-level prevalence

Clinical and forensic interpretation

The most immediate practical value of recognizing variability at the body-greater cornu junction is to avoid misclassifying a persistent developmental interface as acute trauma. A smooth, corticated line in the expected anatomical location, particularly in the absence of displacement or adjacent soft-tissue injury, is more compatible with a persistent developmental junction. By contrast, an irregular noncorticated line, fragment displacement, or associated soft-tissue injury should raise suspicion for fracture [9, 17]. On postmortem CT, osseous disruption can be detected with good diagnostic accuracy, but assessment of subtle fissures and evidence of antemortem injury may still require correlation with dissection and, where appropriate, histology [17]. Fusion status should not be used as a stand-alone chronological marker. Modern CT studies indicate that distant nonfusion, when defined using a specific operational threshold, is predominantly associated with younger individuals, whereas ordinary nonfusion may persist into advanced age and complete fusion may occur in early adulthood [8, 13, 15, 16, 24]. Fusion status may therefore contribute to broad age assessment but cannot reliably define a narrow chronological interval. Age estimation should integrate junctional morphology with other validated skeletal, dental, or imaging indicators rather than infer age from a single hyoid feature. Similar caution is required in sex estimation. Contemporary systematic evidence supports measurable sexual dimorphism of the hyoid and reports promising performance of multivariate and machine-learning approaches based on morphometric variables [19]. However, population heterogeneity, age distribution, and variability in fusion status limit generalizability. Three-dimensional geometric morphometrics can characterize overall hyoid shape [21], but these methods address a different biological question from histological characterization of the body-greater cornu interface. Neither morphometric nor artificial-intelligence classification should therefore be interpreted as evidence that an imaging-defined fusion state corresponds to histologically complete synostosis. Pollanen and Chiasson compared fractured and unfractured hyoids from victims of homicidal strangulation and observed more frequent ossification or fusion among fractured specimens [18]. However, the fractured group was older and also differed in hyoid morphology. Their findings therefore support, at most, a possible association among age, skeletal rigidity, morphology, and fracture susceptibility; they do not establish fusion as an independent causal determinant of fracture. For surgical purposes, the body-greater cornu junction should be regarded primarily as an anatomical variable. Recognition may be relevant during imaging or procedures involving the suprahyoid region, floor of the mouth, and upper neck. Synostosis could plausibly reduce junctional mobility and alter local load transmission, but this has not been demonstrated clinically. Available studies have not established an association between fusion status and the technique, complications, or outcome of hyoid suspension, upper-neck reconstruction, or floor-of-mouth surgery. Its surgical significance should therefore be described cautiously and not presented as predictive.

Evidence gaps and hypothesis

The principal unresolved biological question is whether the range of adult junctional phenotypes reflects a common remodeling pathway with variable timing, several persistent anatomical phenotypes, or a combination of these possibilities. Available histological evidence is cross-sectional: different individuals are examined at different ages, and no longitudinal human study has followed an individual body-greater cornu junction from a cartilaginous state through mineralization and eventual synostosis [5-7]. Accordingly, the histological grades described by Kanetaka et al. should be interpreted as cross-sectional states rather than demonstrated sequential stages. A developmental continuum remains a plausible hypothesis, but it has not been established as an obligatory biological pathway. Local mechanical loading represents another hypothesis rather than an established determinant. The body and greater cornua have different muscular and fascial attachments, and micro-CT studies demonstrate regional differences in hyoid trabecular architecture [1, 28]. These observations provide a biological rationale for load-responsive remodeling. Nevertheless, no junction-specific longitudinal human study or validated biomechanical model has demonstrated that swallowing mechanics, muscular traction, or local force transmission causes body-greater cornu synostosis. Mechanical influences should therefore be investigated experimentally rather than invoked as an explanation for observed age-related patterns. Other potentially relevant factors, including systemic bone metabolism, endocrine status, previous trauma or microtrauma, and population-related variation, remain poorly studied. Future research should evaluate these variables using designs capable of separating their effects from those of age. At present, they should be regarded as evidence gaps or testable hypotheses rather than established determinants of junctional fusion.

Limitations

This review has several methodological limitations. It is a structured narrative review with critical appraisal, and the literature search was not prospectively recorded at the level required for a PRISMA systematic review. Exact numbers of records retrieved, deduplicated, screened, and excluded cannot be reconstructed reliably, and no claim of PRISMA compliance is made. Study selection was not performed through a formal duplicate-independent screening workflow, and no single risk-of-bias instrument was applied across all included studies. Although the revised Methods provide explicit search concepts, eligibility criteria, evidence-extraction fields, and study-level appraisal domains, a prospectively registered systematic review would offer greater reproducibility.

The underlying literature is also highly heterogeneous. Studies differ in their definitions of fusion, age stratification, populations, imaging or histological methods, and use of side-level versus person-level denominators. Several samples are small or population-specific, and many reported sex associations have not been adjusted adequately for age, body size, or other potential confounders. Furthermore, routine clinical CT cannot determine microscopic cartilage composition or marrow continuity, creating the possibility of discordance between imaging-defined fusion and histologically confirmed synostosis.

The independence of some cadaveric histological datasets is also uncertain. The Shimizu and Kanetaka studies share investigators and arise from closely related cadaveric research, but the extent of specimen overlap cannot be determined from the published reports [6,7]. Their prevalence findings are therefore not treated as independent datasets or quantitatively combined. Finally, the absence of longitudinal human evidence remains the principal biological limitation: currently available studies can demonstrate age-associated differences in the distribution of junctional states, but they cannot establish the natural history of an individual junction.

Discussion

The available evidence supports a more nuanced interpretation of the body-greater cornu junction than a simple fused/nonfused model. Several conclusions can be stated with reasonable confidence. First, direct human embryological studies demonstrate a median hyoid-body condensation that is morphologically distinct from the lateral greater-cornu precursor, although these observations do not establish cellular lineage [2, 3]. Second, ossification of the individual hyoid components is biologically distinct from subsequent remodeling and possible union of the intervening junction [4, 8, 14, 15]. Third, adult histology demonstrates genuine tissue-level diversity, including hyaline and fibrocartilage, clefts or joint-like spaces, peripheral mineralization, incomplete osseous bridging, and complete synostosis [5-7]. Fourth, more advanced forms of junctional union become increasingly frequent with age, but fused and nonfused states overlap substantially across adulthood and do not support a fixed chronological threshold [7, 8, 10-16, 22-26]. Much of the apparent disagreement among previous studies can be explained by endpoint heterogeneity.

A radiographic junctional line, a CT-defined gap threshold, peripheral mineralization, incomplete osseous bridging, unilateral person-level fusion, and histologically confirmed complete synostosis are biologically and methodologically distinct findings. When these different endpoints are grouped under the single term fusion, variation in reported prevalence and age associations becomes difficult to interpret. The same problem affects reported sex and laterality differences, which may reflect differences in age composition, study population, imaging technique, operational definition, or analytical denominator rather than stable biological effects.

The most informative next step is therefore not simply another large prevalence study. A more definitive investigation would recruit or assemble an age-balanced human sample and apply prespecified thin-section CT with multiplanar assessment, followed-where feasible in cadaveric material-by micro-CT and blinded histological examination of the same body-greater cornu junctions. Imaging categories should be defined prospectively and validated against tissue-defined endpoints. Each side should be recorded separately while preserving person-level laterality patterns, and statistical analyses should account for within-person correlation between paired junctions. Interobserver agreement should be quantified, and specimen provenance should be documented clearly to identify repeated use of material across publications.

The proposed four-category operational framework is intended to facilitate such validation. It separates an open/nonfused junction from mineralization without bridging, partial osseous bridging, and complete osseous synostosis, while treating unilateral or bilateral fusion as person-level descriptors. The framework is intended to improve transparency and comparability rather than establish new consensus nomenclature. Its reproducibility, imaging-histology validity, and clinical utility should therefore be evaluated before wider adoption.

## Conclusions

The human body-greater cornu junction is a variable developmental interface rather than a fixed chronological marker. Ossification of the individual components, remodeling or mineralization of the intervening junction, partial osseous bridging, and complete synostosis represent distinct biological observations and should not be collapsed into a single binary definition of fusion. Cross-sectional evidence demonstrates an age-associated shift toward greater junctional union, but complete synostosis may occur in young adults and nonfusion may persist into advanced age; the available evidence does not demonstrate an obligatory sequential pathway. Radiographic approximation, sclerosis, or marginal mineralization should not automatically be equated with histologically complete osseous continuity. Reports of fusion should specify the operational definition, imaging or histological method, laterality, and unit of analysis. The proposed four-category operational framework provides a provisional and testable basis for more consistent reporting but requires validation before it can be regarded as consensus terminology. Future research should prioritize same-specimen correlation of standardized thin-section CT, micro-CT, and histology in independently documented, age-balanced human material, with assessment of interobserver agreement and explicit distinction between side-level morphology and person-level fusion patterns.

## References

[1] Standring S, Tubbs RS (2025). Gray’s Anatomy: the anatomical basis of clinical practice. 43rd ed.. Gray’s Anatomy: The Anatomical Basis of Clinical Practice. 43rd ed. Elsevier.

[2] Rodríguez-Vázquez JF, Kim JH, Verdugo-López S, Murakami G, Cho KH, Asakawa S, Abe S (2011). Human fetal hyoid body origin revisited. J Anat.

[3] de Bakker BS, de Bakker HM, Soerdjbalie-Maikoe V, Dikkers FG (2018). The development of the human hyoid-larynx complex revisited. Laryngoscope.

[4] Reed MH (1993). Ossification of the hyoid bone during childhood. Can Assoc Radiol J.

[5] Koebke J, Saternus KS (1979). Morphology of the adult human hyoid bone (Article in German). Z Rechtsmed.

[6] Shimizu Y, Kanetaka H, Kim YH, Okayama K, Kano M, Kikuchi M (2005). Age-related morphological changes in the human hyoid bone. Cells Tissues Organs.

[7] Kanetaka H, Shimizu Y, Kano M, Kikuchi M (2011). Synostosis of the joint between the body and greater cornu of the human hyoid bone. Clin Anat.

[8] Fisher E, Austin D, Werner HM, Chuang YJ, Bersu E, Vorperian HK (2016). Hyoid bone fusion and bone density across the lifespan: prediction of age and sex. Forensic Sci Med Pathol.

[9] MacDonald-Jankowski DS (1990). The synchondrosis between the greater horn and the body of the hyoid bone: a radiological assessment. Dentomaxillofac Radiol.

[10] O'Halloran RL, Lundy JK (1987). Age and ossification of the hyoid bone: forensic implications. J Forensic Sci.

[11] Miller KW, Walker PL, O'Halloran RL (1998). Age and sex-related variation in hyoid bone morphology. J Forensic Sci.

[12] Jadav D, Shedge R, Kanchan T, Meshram V, Garg PK, Krishan K (2022). Age-related changes in the hyoid bone: an autopsy-based radiological analysis. Med Sci Law.

[13] Abbasoğlu TT, Göçmen R, Dağ O, Fırat A (2026). Morphometric analysis and a novel classification of the hyoid bone. J Anat Soc India.

[14] Ito K, Ando S, Akiba N (2012). Morphological study of the human hyoid bone with three-dimensional CT images -Gender difference and age-related changes. Okajimas Folia Anat Jpn.

[15] Naimo P, O'Donnell C, Bassed R, Briggs C (2015). The use of computed tomography in determining development, anomalies, and trauma of the hyoid bone. Forensic Sci Med Pathol.

[16] Mutlu GD, Aşırdizer M, Kartal E, Keskin S, Mutlu İ, Göya C (2024). Type and fusion identification by age and sex in human hyoid bone using 3D CT images in a Turkish sample. Eastern J Med.

[17] Hansen JP, Larsen ST, Jacobsen C (2024). Diagnostic accuracy of post-mortem computed tomography for fractures of the hyoid-larynx complex. Int J Legal Med.

[18] Pollanen MS, Chiasson DA (1996). Fracture of the hyoid bone in strangulation: comparison of fractured and unfractured hyoids from victims of strangulation. J Forensic Sci.

[19] Beltran-Aroca CM, Checa-Poza A, De-La-Barrera-Aranda E, Girela-Lopez E, Trujillo-Mederos A, Romero-Saldaña M (2026). Is there sufficient evidence that the hyoid bone can be used for sexual dimorphism? A systematic review and meta-analysis. Int J Legal Med.

[20] Soerdjbalie-Maikoe V, van Rijn RR (2008). Embryology, normal anatomy, and imaging techniques of the hyoid and larynx with respect to forensic purposes: a review article. Forensic Sci Med Pathol.

[21] Fakhry N, Puymerail L, Michel J (2013). Analysis of hyoid bone using 3D geometric morphometrics: an anatomical study and discussion of potential clinical implications. Dysphagia.

[22] D'Souza DH, Harish SS, Kiran J (2010). Fusion in the hyoid bone: usefulness and implications. Med Sci Law.

[23] Harjeet K, Synghal S, Kaur G, Aggarwal A, Wahee P (2010). Time of fusion of greater cornu with body of hyoid bone in Northwest Indians. Leg Med (Tokyo).

[24] Cundey R, Alcaide CP, Jerković I, Barbir M, Holland MM, Bašić Ž (2026). Analysis of hyoid bone variations to estimate sex, age, and morphology: a study on the Croatian population for forensic applications. Int J Legal Med.

[25] Gupta A, Kohli A, Aggarwal NK, Banerjee KK (2008). Study of age of fusion of hyoid bone. Leg Med (Tokyo).

[26] Balseven-Odabasi A, Yalcinozan E, Keten A (2013). Age and sex estimation by metric measurements and fusion of hyoid bone in a Turkish population. J Forensic Leg Med.

[27] Timmerman G, Van Goethem A, Docter D (2025). Evaluating micro-computed tomography for investigation of the pediatric hyoid-larynx complex. Pediatr Radiol.

[28] Wang X, Wang C, Zhang S (2020). Microstructure of the hyoid bone based on micro-computed tomography findings. Medicine (Baltimore).

